# Supplementary material for: A Critical View of the Application of the APEX Software (Aqueous Photochemistry of Environmentally-Occurring Xenobiotics) to Predict Photoreaction Kinetics in Surface Freshwaters
Source: Molecules. 2019 Dec 18;25(1):9. doi: 10.3390/molecules25010009 (PMC7017383; doi:10.3390/molecules25010009)
Supplement: Supplementary file 1 [file molecules-25-00009-s001.zip › 3.2.4_gcc-4.4.0.zip!/3.2.4_gcc-4.4.0/3.2.4_gcc-4.4.0/doc/octave/html/interpreter/Function-Precedence.html]

Function Precedence - Untitled


Previous: Function Locking,
Up: Function Files


---

#### 11.7.6 Function Precedence

Given the numerous different ways that Octave can define a function, it
is possible and even likely that multiple versions of a function, might be
defined within a particular scope. The precedence of which function will be
used within a particular scope is given by

1. Subfunction
   A subfunction with the required function name in the given scope.- Private function
     A function defined within a private directory of the directory
     which contains the current function.- Class constructor
       A function that constuctors a user class as defined in chapter
       Object Oriented Programming.- Class method
         An overloaded function of a class as in chapter
         Object Oriented Programming.- Legacy Dispatch
           An overloaded function as defined by See doc-dispatch.- Command-line Function
             A function that has been defined on the command-line.- Autoload function
               A function that is marked as autoloaded with See doc-autoload.- A Function on the Path
                 A function that can be found on the users load-path. There can also be
                 Oct-file, mex-file or m-file versions of this function and the precedence
                 between these versions are in that order.- Built-in function
                   A function that is builtin to Octave itself such as `numel`,
                   `size`, etc.
